# A Monoclonal Antibody-Based Time-Resolved Fluorescence Microsphere Lateral Flow Immunoassay for Dinotefuran and Clothianidin Detection

**DOI:** 10.3390/foods14071174

**Published:** 2025-03-27

**Authors:** Lehong Qin, Haojie Chen, Yingxiang Nie, Mengxin Zhou, Junjun Huang, Zhili Xiao

**Affiliations:** Guangdong Provincial Key Laboratory of Food Quality and Safety, College of Food Science, South China Agricultural University, Guangzhou 510642, China

**Keywords:** dinotefuran, clothianidin, monoclonal antibody, time-resolved fluorescence microsphere lateral flow immunoassay

## Abstract

Dinotefuran and clothianidin belong to the third generation of nicotinic insecticides and are widely used in crop pest control. It is necessary to detect their residues in food. The time-resolved fluorescent microspheres lateral flow immunoassay (TRFMs-LFIA) has the advantages of high sensitivity, short duration, and simple operation and is suitable for rapid field testing. In this study, two haptens (FCA-1, FCA-2) were synthesized in three steps and conjugated to the carrier proteins to obtain artificial antigens, which were subsequently used for monoclonal antibody preparation. A TRFMs-LFIA based on monoclonal antibodies was established to detect dinotefuran and clothianidin residues in food. The limit of detection (LOD) for dinotefuran was 0.045 ng/mL, with an IC_50_ of 0.61 ng/mL and a linear range (IC_20_~IC_80_) of 0.12~3.11 ng/mL. The LOD for clothianidin was 0.11 ng/mL, with an IC_50_ of 0.94 ng/mL and a linear range (IC_20_~IC_80_) of 0.24~3.65 ng/mL. Cross-reactivity rates with seven tested structural analogs were less than 1.5%. The pretreatment method was optimized for wheat, cucumber, and cabbage samples, which was time-saving (20 min) and easy to operate. The average recovery rates ranged from 88.0% to 114.8%, with the corresponding coefficients of variation appearing (CV) between 1.9% and 13.5%. The results of actual wheat, cucumber, and cabbage samples detected by the established TRFMs-LFIA were consistent with those of Ultra-Performance Liquid Chromatography coupled with Tandem Mass Spectrometry (UPLC-MS/MS). These results demonstrate that the established TRFMs-LFIA is sensitive, accurate, rapid, and suitable for real sample detection.

## 1. Introduction

Dinotefuran and clothianidin are classified as third-generation nicotinic insecticides and were registered in 2002 [1] and 2001 [2], respectively, in Japan. These insecticides possess a broad insecticidal spectrum, low toxicity, and high efficiency, making them widely utilized globally [3]. They all contain 3-methyl-2-nitroguanidinium and lack an aromatic group compared with other nicotinic pesticides. They mainly paralyze and kill the insects by affecting nicotinic acetylcholine receptors in the central nervous system of these organisms [4].

In recent years, an increasing number of studies have investigated the detrimental effects of neonicotinoid pesticides on non-target animals. Relevant studies have reported that both dinotefuran and clothianidin are harmful to aquatic organisms [5,6] and amphibians [7,8]. Several papers have shown damage to the liver, intestines, and reproductive system caused by neonicotinoid pesticides, including dinotefuran and clothianidin [9].

Several countries have established standards to specify the maximum residue limits for dinotefuran and clothianidin in food. For example, the European Union, the United States, and South Korea have set the residue limits for dinotefuran in garlic, ginger, and tea at 0.01, 0.05, and 7 mg/kg, respectively, while the limits for clothianidin are 0.01, 0.3, and 0.6 mg/kg, respectively [10,11,12]. In China, the maximum residue limits for dinotefuran and clothianidin in food range from 0.01 to 20 mg/kg [13].

It is crucial to detect dinotefuran and clothianidin residues in food. Current detection methods (Table 1) primarily rely on instrumental analysis, such as High-Performance Liquid Chromatography (HPLC), High-Performance Liquid Chromatography coupled with Tandem Mass Spectrometry (HPLC-MS/MS), and Ultra-Performance Liquid Chromatography coupled with Tandem Mass Spectrometry (UPLC-MS/MS). These methods depend on sophisticated instruments that offer high accuracy but often require professional operation, incur high costs, and involve complex processes, making them unsuitable for rapid detection. In contrast, an immunoassay based on the specific binding of antigens and antibodies is typically more convenient and faster.

Zhao et al. [14] established an immunocapture enzyme-linked immunosorbent assay (icELISA) to detect dinotefuran residues based on monoclonal antibodies, and the IC_50_ was 5.66 ng/mL. Uchigashima et al. [15] established a direct competition ELISA for clothianidin detection based on monoclonal antibodies, with an IC_50_ of 4.4 ng/mL, and found that the cross-reactivity rate with dinotefuran was 64%. Chang et al. [16] established the icELISA method for clothianidin detection based on the recombinant antibody with an IC_50_ of 4.62 ng/mL. Li [2] established an icELISA for clothianidin detection based on three types of antibodies (polyclonal, monoclonal, and single-chain antibodies), with an IC_50_ of 46, 25.6, and 62.3 ng/mL, respectively, and found that the cross-reactivity rates with dinotefuran were 11.8%, 46.5%, and 51.9%, respectively. An et al. [17] developed a colloidal gold strip based on monoclonal antibodies to detect dinotefuran residues, with a detection limit of 0.5 mg/kg. Li et al. [18] developed an immunochromatographic assay (ICA) to detect clothianidin residues based on monoclonal antibodies, and the lowest cut-off limit was 4 ng/mL. Li [2] established the time-resolved fluorescence immunoassay (TRFIA) and fluorescence polarization immunoassay (FPIA) to detect clothianidin residues based on monoclonal antibodies, with an IC_50_ of 2.07 and 87.3 ng/mL, respectively. Xu et al. [19] constructed a competitive fluorescent microsphere suspension immune sensor to detect residues of neonicotinoid pesticides, and the detection limit of dinotefuran was 95 ng/mL.

**Table 1:** Detection methods of dinotefuran or clothianidin.

Methods		Detection Target	IC_50_ (ng/mL)	LOD (ng/mL)	Detection Time (min)	References
Instrumental analysis	UPLC-MS/MS	Dinotefuran	-	0.04	-	[20]
	UPLC-MS/MS	Clothianidin		5	-	[21]
	UPLC	Clothianidin		52.7	-	[22]
Immunoassay	IcELISA	Dinotefuran	5.66	-	>60	[14]
	DcELISA	Clothianidin	4.4	-	>60	[15]
	IcELISA	Clothianidin	4.62	-	>60	[16]
	IcELISA	Clothianidin	25.6	-	>60	[2]
	ICA	Clothianidin	-	4	5	[18]
	TRFIA	Clothianidin	2.07	-	-	[2]
	FPIA	Clothianidin	87.3	-	-	[2]

According to Table 1, the most common immunoassay method for the detection of dinotefuran or clothianidin is ELISA, but it has the disadvantages of being time-consuming and cumbersome. Both ICA and FPIA have low sensitivity. TRFIA utilizes lanthanide chelates as markers, and its sensitivity still has the potential for enhancement. However, the time-resolved fluorescent microspheres lateral flow immunoassay (TRFMs-LFIA) is faster and simpler. TRFMs-LFIA is an immunoassay that combines fluorescence and chromatography, utilizing time-resolved fluorescent microspheres as markers that bind multiple lanthanide chelates on microspheres [23], making it more sensitive than TRFIA. Compared to traditional fluorescent materials, lanthanides offer several advantages, including long fluorescence lifetimes, a large Stokes shift between excitation and emission wavelengths, and a broad excitation spectrum coupled with a narrow emission spectrum. These properties make TRFMs-LFIA less susceptible to interference from background fluorescence and scattered light, resulting in enhanced stability and sensitivity. TRFMs-LFIA is faster compared to ELISA and more sensitive than TRFIA. Therefore, TRFMs-LFIA has been widely used in clinical testing, in vitro diagnostics, agriculture, veterinary drug residue detection, drug screening, environmental monitoring, and various other fields.

Several publications have reported the application of TRFMs-LFIA in pesticide residue detection. Cheng et al. [24] established a sensitive and rapid TRFMs-LFIA based on monoclonal antibodies with an IC_50_ of 9.38 ng/mL. Chen et al. [25] established a highly sensitive TRFMs-LFIA for the detection of propanonazole, and the quantitative detection limit was 1.92 ng/mL. Deng et al. [26] established a TRFMs-LFIA for the rapid quantitative detection of cabendazim within 10 min with a detection limit of 0.41 ng/mL and 0.56 ng/mL for rice and tobacco, respectively. Xu et al. [27] developed a TRFMs-LFIA for the detection of thiacloprid with a detection limit of 0.003 ng/mL and a recovery rate between 79.4% and 118.6%.

In this study, TRFMs-LFIA simultaneously detected dinotefuran and clothianidin residues in food based on monoclonal antibodies. With high sensitivity, short testing times, simple operation, and convenient storage and transportation, it can achieve real-time fast and sensitive detection.

## 2. Materials and Methods

### 2.1. Materials and Instruments

Keyhole limpet hemocyanin (KLH), ovalbumin (OVA), polyethylene glycol (PEG) 1450, complete and incomplete Freund’s adjuvants, goat anti-mouse immunoglobulin G (IgG), hypoxanthine–thymidine (HT), and hypoxanthine–aminopterin–thymidine (HAT) were bought from Sigma-Aldrich Chemical Co., Ltd. (St. Louis, MO, USA). Concanavalin A (CONA) was bought from Yuanye Bio-Technology Co., Ltd. (Shanghai, China). Lactoferrin (LF) was bought from Wako Pure Chemical Industries Co., Ltd. (Osaka, Japan). Bovine serum albumin (BSA) was bought from Biofroxx Co., Ltd. (Essen, Germany). 2-(N-morpholino) ethane sulfonic acid (MES) and 2-[4-(2-hydroxyethyl) piperazin-1-yl] ethanesulfonic acid (HEPES) were bought from Macklin Biochemical Co., Ltd. (Shanghai, China). RPMI-1640 culture media were purchased from ExCell Bio Co., Ltd. (Suzhou, China). Fetal bovine serum (FBS) and the pierce rapid isotyping kit-mouse were purchased from Thermo Fisher Scientific Co., Ltd. (Waltham, MA, USA). The time-resolved fluorescence microsphere (TRFM), at 0.2 μm, was obtained from Bangs Laboratories, Inc. (Fishers, IN, USA). Standards (purity ≥ 98%) of dinotefuran, clothianidin, acetamiprid, nitenpyram, diuron, thiacloprid, tetrahydrofuran, thiamethoxam, and imidacloprid were purchased from TM Standard Co., Ltd. (Beijing, China). All organic solvents and chemicals used were of analytical pure grade.

Nitrocellulose (NC) membranes (Sartorius, UniSart CN 140) were purchased from Sartorius Stedim Biotech GmbH (Goettingen, Germany). Cell culture dishes (6 cm and 10 cm) and cell culture plates (6, 12, 24, and 96-well) were purchased from Corning Incorporated (Corning, USA). The sample pads (RB65), PVC backing plates (SMA31-40), and absorbent pads (CH37K) were purchased from Shanghai Liangxin Co., Ltd. (Shanghai, China). The NanoDrop 2000c UV-Vis spectrophotometer was purchased from Thermo Fisher Scientific Co., Ltd. (Waltham, MA, USA). The liquid chromatograph–triple quadrupole mass spectrometer was provided by AB SCIEX Analytical Instrument Trading Co., Ltd (Boston, MA, USA). The FIC-Q1 multifunctional fluorescence reader was obtained from Fenghang Technology Co., Ltd. (Hangzhou, China). The Zetasizer Nano ZS90 was supplied by Malvern Panalytical (Malvern, UK). Balb/c mice were purchased from Zhuhai BesTest Bio-Tech Co., Ltd. (Zhuhai, China). Wheat, cucumber, and cabbage samples were all bought from a farmers’ market in Guangzhou, China.

### 2.2. Methods

#### 2.2.1. Synthesis of Haptens and Antigens

**Figure 1:**
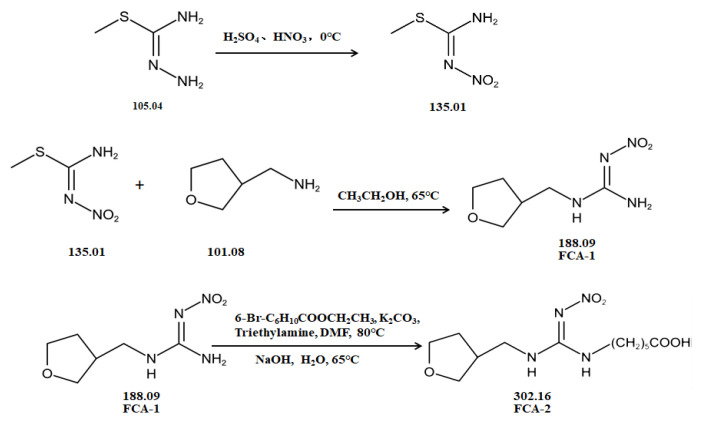
The synthetic route of haptens (FCA-1, FCA-2).

Two haptens, termed FCA-1 and FCA-2, were synthesized (Figure 1) according to reported studies with some modifications [28,29]. The two haptens were then coupled to carrier proteins by glutaraldehyde and active ester methods, respectively, to prepare artificial antigens. Solution A was made by mixing 0.12 mmol OVA and 0.02 mmol glutaraldehyde in a CBS buffer (0.01 M, pH 9.6) and was stirred at 4 °C for 30 min. Solution B was made by dissolving 0.02 mmol FCA-1 in 300 μL DMF. Then, solution B was slowly dropped into solution A, and the mixed solution was stirred at 4 °C for 8 h; it was then terminated by adding sodium borohydride to obtain solution C.

Solution D was made by mixing 0.01 mmol FCA-2, 0.015 mmol EDC, and 0.015 mmol NHS in 1 mL DMF and stirring overnight at 4 °C. Solution E was made by dissolving carrier proteins (OVA, BSA, KLH, LF OR OVA) in a 3 mL CBS buffer (0.01 M, pH 9.6). Solution F was obtained by slowly dropping solution D into solution E, which was stirred at 4 °C for 12 h.

The conjugates (FCA-1-OVA, FCA-2-OVA, FCA-2-BSA, FCA-2-KLH, FCA-2-LF, FCA-2-CONA) were obtained by dialyzing solutions C and F in the PBS buffer for 3 d at 4 °C, respectively, and this was characterized by UV-Vis spectroscopy.

#### 2.2.2. Preparation of Monoclonal Antibodies

Seven-week-old female Balb/c mice were injected with 200 μL of a mixture made by emulsifying Freund’s adjuvant with 1 mg/mL immunogen (FCA-2-BSA, FCA-2-KLH, FCA-2-LF or FCA-2-CONA) at a 1:1 volume ratio subcutaneously and intraperitoneally. Serum was taken from the tail vein after the third immunization and tested by icELISA with FCA-1-OVA or FCA-2-OVA as the coating antigen. Serum from the Balb/c mouse immunized with FCA-2-KLH presented better titer and inhibition rates for dinotefuran. Therefore, this mouse was used for the preparation of hybridoma.

The procedures for hybridoma preparation are as follows: mouse splenocytes obtained by homogenizing the spleen were mixed evenly and subjected to cell fusion with SP2/0 cells in the presence of PEG 1450. The fused cells were dispersed in an HAT medium and were then cultured in 96-well plates. Cell supernatants were detected by icELISA. A limited dilution was used to screen the cells. Ascites was induced by injecting hybridoma into Balb/c mice intraperitoneally purified by Protein G affinity chromatography. The purity and subtype of the monoclonal antibody (mAb) were identified by sodium dodecyl sulfate-polyacrylamide gel electrophoresis (SDS-PAGE) and the mouse antibody isotype kit, respectively.

#### 2.2.3. Establishment of TRFMs-LFIA

1. Preparation of TRFM probes

The principle of probe preparation is the covalent coupling of the amino on mAbs to the carboxyl on TRFMs. The preparation process was performed as follows [24]. The TRFMs were dispersed in an MES buffer (0.05 M); then, EDC was added, and the mixture was shaken for 15 min to activate the carboxyl groups of the TERMs. The sediments retained after centrifugation were dissolved in a BB (0.02 M) with a moderate amount of anti-dinotefuran mAbs (1 mg/mL), and the mixture was shaken for 1 h. Then, the unbound active sites of TRFMs were blocked with BSA. Finally, the prepared probes were dispersed in PB (0.02 M) containing 0.5% (*w*/*v*) BSA, 0.3% (*w*/*v*) PVP, 0.5% (*v*/*v*) Tween-20, 5% (*w*/*v*) sucrose, and 0.03% (*w*/*v*) Proclin-300. The probes were characterized by Zetasizer Nano ZS90.

2. Fabrication of TRFMs-LFIA strips

**Figure 2:**
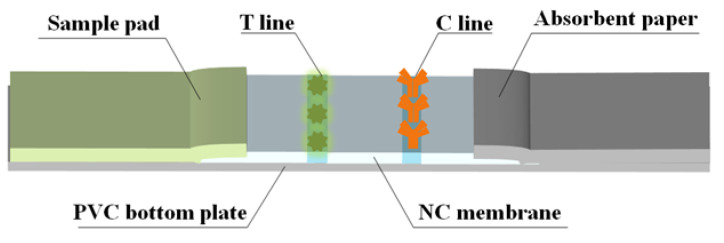
Fabrication of TRFMs-LFIA strips.

The strip consists of a PVC bottom plate, sample pad, NC membrane, and absorbent paper. The initial treatment solution for the sample pad used a PB (0.2 M) containing 0.3% (*w*/*v*) PVP and 0.5% (*v*/*v*) Tween-20. The T line and C line of the NC membrane were coated with FCA-1-OVA and a goat anti-mouse IgG antibody, respectively. The processed sample pad and NC membrane were dried at 50 °C and 37 °C for 12 h and then assembled, as shown in Figure 2. The cut strips were sealed at room temperature under dry conditions.

3. Detection process of TRFMs-LFIA

**Figure 3:**
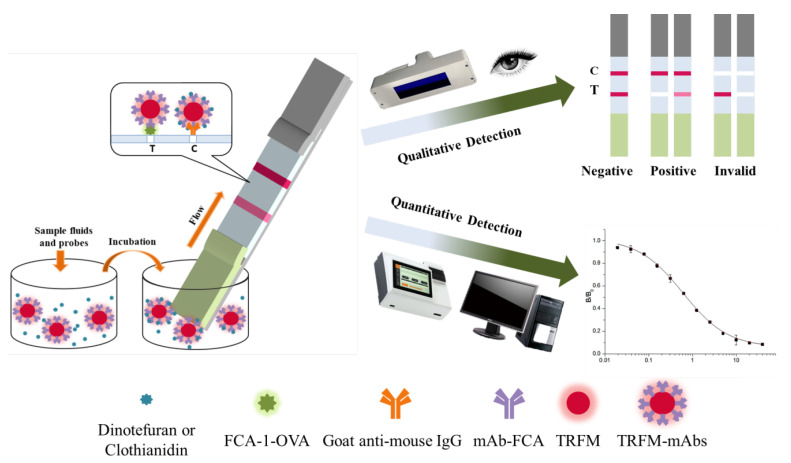
The detection steps and results of the TRFMs-LFIA test strips.

The detection process of TRFMs-LFIA is shown in Figure 3. The standard solution of the target drug or sample treatment solution, mixed with the probes, was added into a microwell. The target drug in the liquid was bound specifically to mAb on a probe. After 5 min, a strip was inserted into the microwell from the sample pad end and then probes flowed upward on the strip. Probes without bonding target drugs were combined with FCA-1-OVA on the T line, while probes with bonding target drugs continued to flow upward and were captured by the goat anti-mouse antibody on the C line. Qualitative analysis can be performed by visual observation under UV light, while quantitative analysis can be performed by an FIC-Q1 multifunctional fluorescence reader. The fluorescence intensity of the T or C line, which correlates with the number of probes, can be measured by the FIC-Q1 multifunctional fluorescence reader. As the concentration of the target drug in the sample increases, the fluorescence intensity ratio of the T to C line decreases. Therefore, it can be judged whether the sample is negative or positive based on the observed fluorescence intensity ratio.

4. Evaluation of different working conditions

To achieve optimal sensitivity, the following parameters of the TRFMs-LFIA were optimized: the number of mAbs, the pH value of the MES buffer used for fluorescent microsphere activation, the number of probes, and the standard dilution. The strip tests were conducted under the above indicators of different gradients; then, the background fluorescence intensity and the T/C line fluorescence intensity were measured with the FIC-Q1 multifunctional fluorescence reader, and inhibition rates were calculated to determine the optimal parameter.

#### 2.2.4. Evaluation of TRFMs-LFIA

1. Evaluation of sensitivity

The standards of dinotefuran and clothianidin were gradient-diluted and tested under optimal conditions. B and B0 represent fluorescence intensity ratios of T to C with and without a standard, respectively. The standard curve was fitted by origin 9.0 using the standard concentration as the *x*-axis and values of B/B0 as the *y*-axis to obtain the limit of detection (LOD), 50% inhibitory concentration (IC_50_), and linear range (IC_20_~IC_80_). The sensitivity of TRFMs-LFIA was evaluated using these parameters.

2. Evaluation of specificity

Seven structural analogs of dinotefuran and clothianidin (tetrahydrofuran, diuron, acetamiprid, nitenpyram, thiacloprid, thiamethoxam, imidacloprid) were diluted by the gradient and tested by TRFMs-LFIA, and standard curves were fitted by origin 9.0 to obtain the IC_50_. Dinotefuran was set as the representative target compound to evaluate the specificity of TRFMs-LFIA. Cross-reaction rates were calculated, respectively, as follows: CR (%) = IC_50_ (dinotefuran, ng/mL)/IC_50_ (structural analog, ng/mL) × 100.

3. Evaluation of stability

The standard of dinotefuran (8 ng/mL) was detected by strips kept at room temperature under dry conditions on days 1, 7, 15, and 30 d, respectively, and the inhibition rates and fluorescence intensity were compared to evaluate the stability of TRFMs-LFIA.

4. Evaluation of accuracy
(1) Elimination of matrix effect

Blank wheat, cucumber, and cabbage samples were detected by HPLC-MS/MS and were identified to be dinotefuran- and clothianidin-free. The matrix effect was eliminated through pretreatment and gradient dilution. The pretreatment method was improved according to the literature [17]. In total, 10 g (±0.01 g) homogenized samples was mixed with a 20 mL PB (0.05 M) containing 10% (*v*/*v*) ethanol in a centrifuge tube. Each tube was shaken for 15 min, and the extract was filtered. The filtrate was diluted in PB (0.05 M) to 1-, 2-, 4-, and 8-fold and tested by TRFMs-LFIA to determine the optimal dilution of the fluorescence intensity close to the PB.

(2) Recovery test

Standards of dinotefuran or clothianidin at high, medium, and low concentrations (within the linear detection range of TRFMs-LFIA) were added to the blank sample, respectively. Each sample was pretreated as described above and tested by TRFMs-LFIA. The test values were calculated according to the standard curve to obtain recovery rates (R, %) and coefficients of variation (CV, %).

#### 2.2.5. Real Sample Detection

Three real samples of wheat, cucumber, and cabbage purchased from the market were pretreated and tested by TRFMs-LFIA and UPLC-MS/MS. The UPLC-MS/MS was analyzed using the Thermo Accucore AQ column. The mobile phases were methanol (A) and aqueous solutions (B), each containing 5 mM ammonium formate and 0.1% (*v*/*v*) formic acid. The gradient elution procedure was programmed as follows: 0–9 min, 90% B—50% B; 9–20 min, 50% B—35% B; 20–30 min, 35% B—0% B; 30–34 min, and 0–90% B.

## 3. Results and Discussion

### 3.1. Synthesis of Haptens and Antigens

**Figure 4:**
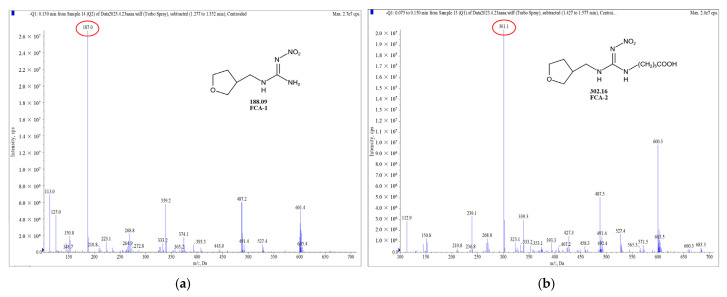
Mass spectrum plot of haptens: (**a**) FCA-1; (**b**) FCA-2.

**Figure 5:**
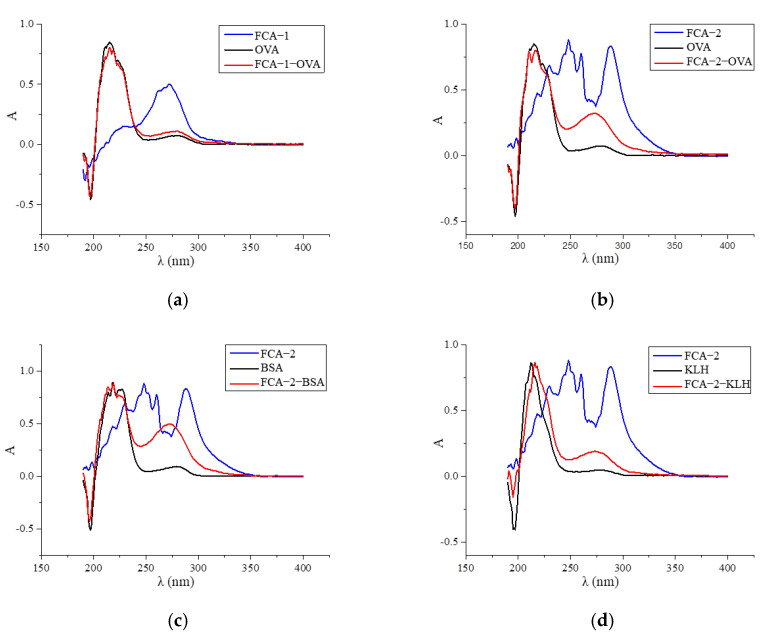

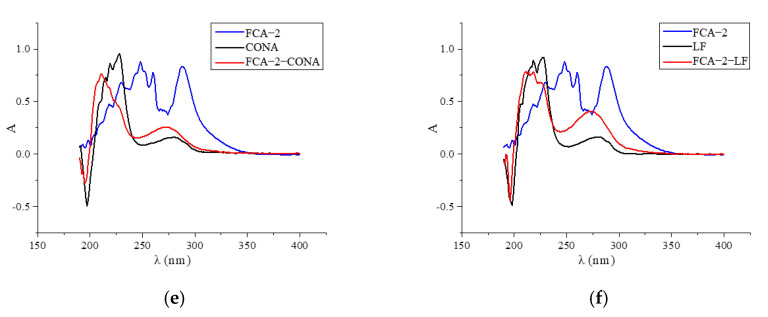
UV scan spectrum of artificial antigens: (**a**) FCA-1-OVA; (**b**) FCA-2-OVA; (**c**) FCA-2-BSA; (**d**) FCA-2-KLH; (**e**) FCA-2-CONA; and (**f**) FCA-2-LF.

Two haptens were synthesized and identified by LC-MS. The *m*/*z* ratios of FCA-1 and FCA-2 were 187.0 and 301.1, respectively, and were obtained by ESI-MS with the negative ion mode (Figure 4), which was consistent with the expected results. Antigens were identified by the UV-Vis spectrum, and the results showed that each antigen absorption front showed clear differences with the hapten as well as the carrier protein around 280 nm (Figure 5). Because of the structural differences between haptens and carrier proteins, they tend to display different characteristic absorption peaks in the UV-Vis spectrum. Consequently, the UV-Vis spectra of the antigens are distinct from those of haptens and carrier proteins.

In this study, the synthesis method is based on two chemical raw materials and three steps of synthesis (Figure 1). In contrast, the synthesis method of the similar haptens reported [17] requires seven main materials, including flammable and explosive reagents, such as formaldehyde, methylamine, and hydrazine hydrate, and involves eight synthetic procedures. Therefore, the synthesis method in this study was simpler, safer, and cost-effective.

### 3.2. Preparation and Characterization of mAb

Serum from Balb/c mice immunized with FCA-2-KLH after the fifth immunization was tested by icELISA with FCA-1-OVA as the coating antigen. The results exhibited a titer of 1:8000 and an inhibition rate of 80% for 1 μg/mL dinotefuran.

**Figure 6:**
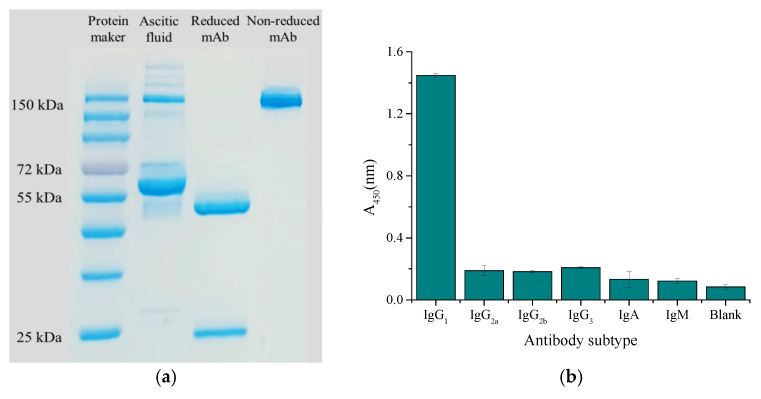
Characterization of antibody: (**a**) SDS-PAGE electropherogram; (**b**) antibody subtype.

Ascites presented multiple different bands on the electropherogram because of its complex composition, while purified mAbs presented a band of about 150 kDa on the electropherogram under non-reduction conditions, and the breakage of disulfide bonds of mAb separated the heavy chain (about 50 kDa) and light chain (about 25 kDa) under reduction condition (Figure 6a). The subtype was identified as IgG_1_ by the murine monoclonal antibody subtype kit (Figure 6b).

In addition, the mAb batch variability is affected by multiple factors in the production process, such as the composition and pH of the cell medium, culture temperature, the stability of hybridoma lines, and purification conditions [30]. The analysis of mAb batch variability can be analyzed by chromatography, Mass Spectrometry, spectroscopy, gel electrophoresis, and other technologies; the principle is to test the structure, purity, and functional activity of mAb [31]. To reduce batch variability, the antibody production process needs strict control and monitoring by ensuring the consistency of the medium and culture temperatures, as well as employing the same purification method [32]. To ensure the consistency of the experiments, all mAbs used in this study were from the same batch.

### 3.3. Establishment of TRFMs-LFIA

#### 3.3.1. Characterization of the TRFM Probes

**Figure 7:**
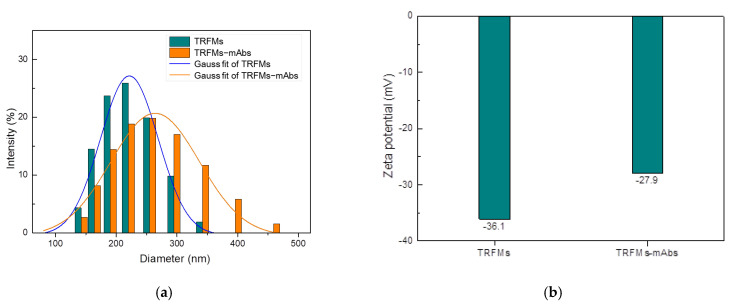
Identification of TRFMs and TRFMs-mAbs: (**a**) particle size; (**b**) Zeta potential.

The characterization of the size and zeta potential of the TRFMs and TRFMs-mAbs was performed by Zetasizer Nano ZS90. The average particle sizes of TRFMs and TRFMs-mAbs were 209.2 nm and 257.0 nm, respectively (Figure 7a), and the average zeta potentials of TRFMs and TRFMs-mAbs were −36.1 mV and −27.9 mV, respectively (Figure 7b), proving that mAbs were successfully coupled with TRFMs.

#### 3.3.2. Evaluation of Different Working Conditions

In this study, probes were prepared by coupling fluorescent microspheres with the mAbs. The pH of the fluorescent microsphere activation buffer and the amount of mAbs were two key factors affecting the probe preparation.

**Figure 8:**
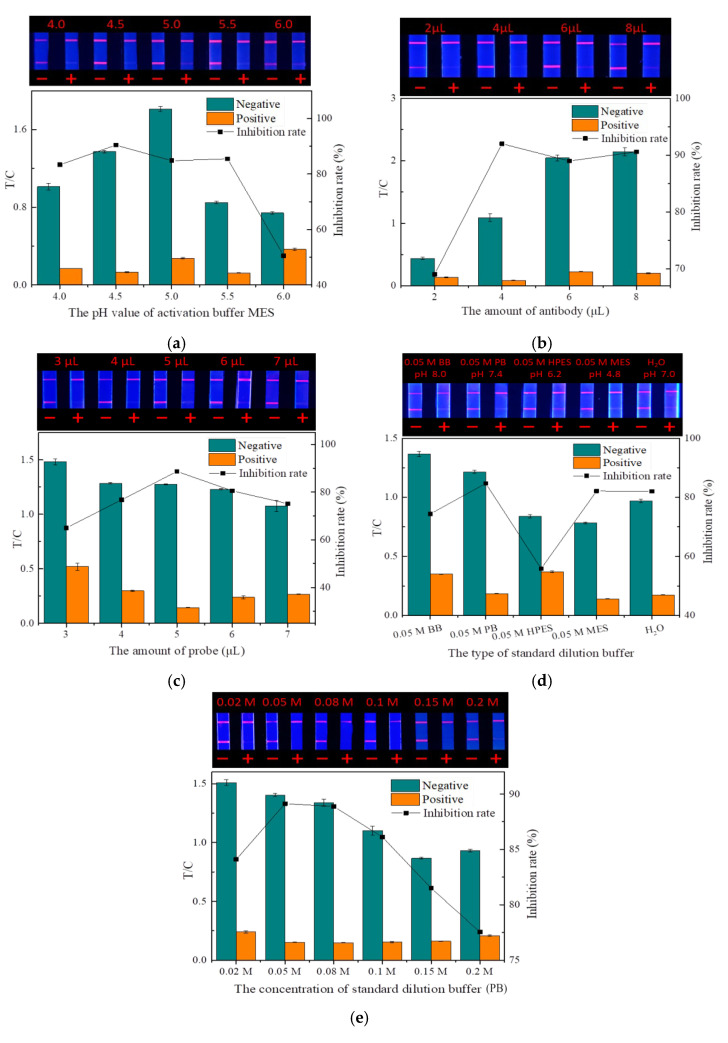
Optimization of the TRFMs-LFIA (*n* = 3): (**a**) the pH value of activation buffer MES; (**b**) the amount of antibody; (**c**) the amount of probe; (**d**) the type of standard dilution buffer; and (**e**) the concentration of standard dilution buffer (PB).

The pH of the microsphere activation buffer (MES) will affect the degree of microsphere activation by affecting the dispersion in the solution [33]. As shown in Figure 8a, when the pH was 4.0, the large aggregation of fluorescent microspheres made activation insufficient, which greatly affected the coupling rate with mAbs, making the fluorescence intensity of both the T and C lines very weak. With the increase in pH, both the fluorescence intensity and inhibition rate showed a trend of first increasing and then decreasing. At the pH value of 4.5, the fluorescence intensity and inhibition effect were optimal, so 4.5 was selected as the best pH for MES.

With a constant number of fluorescent microspheres, both too little and too much mAbs reduced the quality of the probes by reducing the labeling rate and increasing the steric hindrance, respectively [34]. As shown in Figure 8b, the amount of mAbs had a significant effect on the fluorescence intensity of the strip. A small amount of mAbs would cause weak binding between the probe and the T line, causing the low fluorescence intensity of the strip. When the amount of mAbs increased, the fluorescence intensity of the strip and the inhibition rate also increased. When the amount of antibody (1 mg/mL) was 4 µL, the inhibition rate was the highest. With the amount of antibody increasing, the fluorescence intensity continued to increase, but the inhibition rate began to decrease. Therefore, the optimal amount of the antibody added was determined to be 4 µL.

The dosage of probes has a direct influence on the fluorescence intensity and sensitivity of strips. Too little dosage can hinder the normal test, while excessive dosage not only wastes mAbs but also reduces sensitivity and may cause background fluorescence interference, thereby affecting the detection accuracy [35]. The fluorescence intensity of the strip increased significantly with the increasing probe dosage, but when the dosage was greater than 5 μL, the inhibition rate gradually decreased (Figure 8c). Therefore, the optimal dosage of the probes was determined to be 5 μL.

The dispersion state of the drug standard largely depends on the type and concentration of the dilution buffer. Improper buffers can hinder the binding of the mAbs on the probe to the drug. As shown in Figure 8d,e, among the various buffers and ion concentrations, PB (0.05 M, pH 7.4) exhibited the highest inhibition rate and good fluorescence intensity. Therefore, PB (0.05 M, pH 7.4) was selected as the optimal standard dilution.

### 3.4. Evaluation of TRFMs-LFIA

#### 3.4.1. Evaluation of Sensitivity

**Figure 9:**
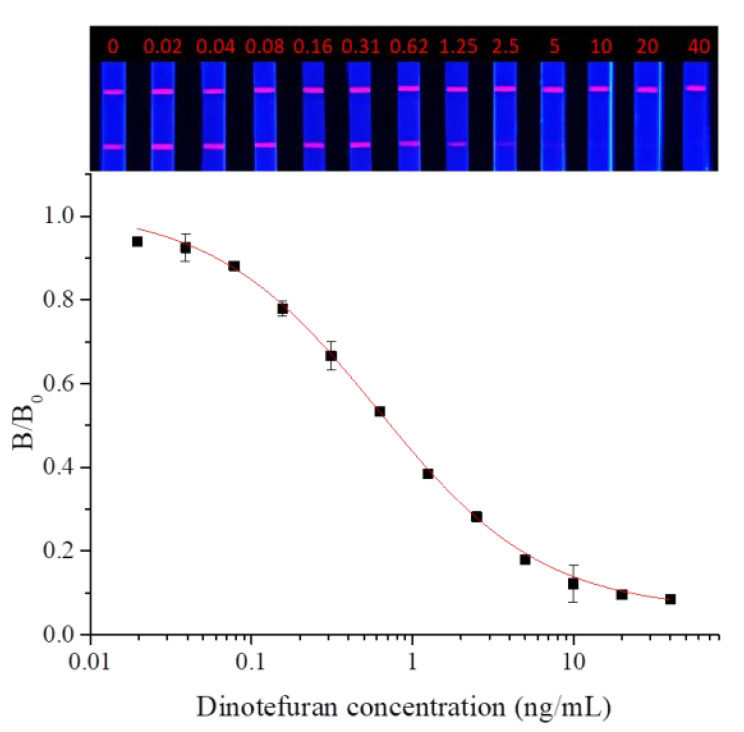
Calibration curve for dinotefuran detection by TRFMs-LFIA (*n* = 3).

**Figure 10:**
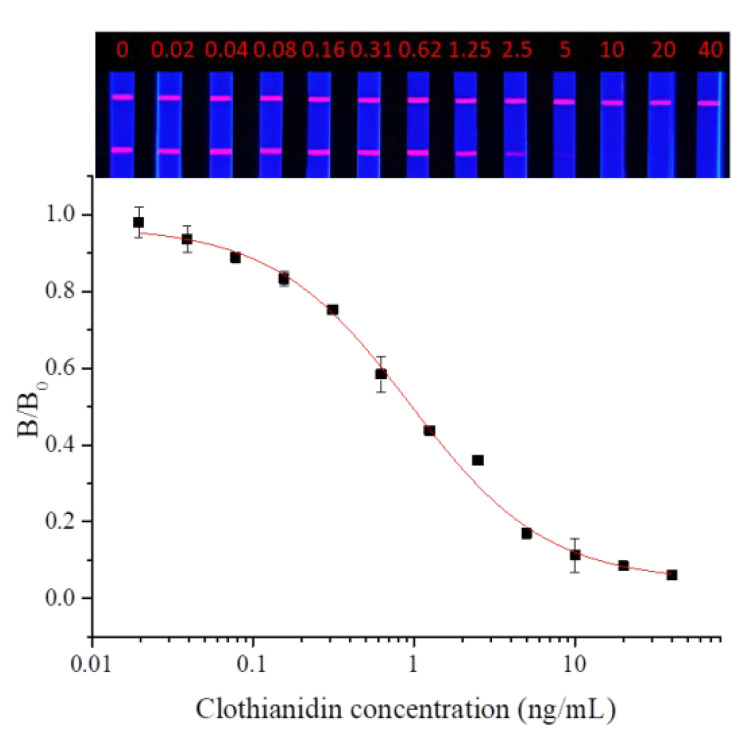
Calibration curve for clothianidin detection by TRFMs-LFIA (*n* = 3).

Dinotefuran and clothianidin standards were diluted to concentrations of 0, 0.02, 0.04, 0.08, 0.16, 0.31, 0.62, 1.25, 2.5, 2.5, 5, 10, 20, and 40 ng/mL. Each dilution was tested by TRFMs-LFIA under the optimal conditions described above. The fluorescence intensity ratio of the T line and C line was measured by the FIC-Q1 multifunctional fluorescence reader. The calibration curve was fitted by origin 9.0. The results are shown in Figure 9 and Figure 10. To dinotefuran, the LOD and the IC_50_ were found to be 0.045 ng/mL and 0.61 ng/mL, respectively, with a linear range (IC_20_~IC_80_) of 0.12~3.11 ng/mL (y = 0.05733 + 0.96237/(1 + (x/0.60632)^0.84775). Toclothianidin, the LOD, and the IC_50_ were determined to be 0.11 ng/mL and 0.94 ng/mL, respectively, with a linear range (IC_20_~IC_80_) of 0.24~3.65 ng/mL (y = 0.04492 + 0.92433/(1 + (x/0.9425)^1.02377). The sensitivity of the established TRFMs-LFIA could meet the detection requirements of GB 2763-2021 (China) and is lower than the reported icELISA [2,14].

#### 3.4.2. Evaluation of Specificity

The cross-reaction rates of the established TRFMs-LFIA were 0.6%, 1.2% with thiacloprid, imidacloprid, and were less than 0.1% with tetrahydrofuran, diuron, acetamiprid, nitenpyram, and thiamethoxam (Table 2). The results show that the established TRFMs-LFIA has a high affinity for dinotefuran and clothianidin but does not cross with tetrahydrofuran, suggesting that the site specifically recognized by the mAb is most likely 3-methyl-2-nitroguanidinium both in dinotefuran and clothianidin. There is relevant literature [2,15] reporting that the method based on mAbs obtained by haptens with an analogous structure can detect these two pesticides.

The established TRFMs-LFIA can be utilized for the initial screening of both dinotefuran and clothianidin due to their high specificity. These two drugs are extremely similar and generally will not be used simultaneously, and further accurate detection can be performed using instrumental methods. Additionally, the simultaneous detection of multicomponent residues can reduce the repeated detection steps and sample consumption, ultimately saving costs and enhancing efficiency. The premise for the establishment of an mAb-based immunoassay is the design of hapten with the common structure of the tested subjects, which greatly affects the specificity and sensitivity of the method [36]. Wang et al. [37] established an ELISA for the simultaneous detection of pyrethroids based on an mAb that recognizes the phenoxybenzene group of pyrethroids. Xu et al. [38] developed an FPIA for the simultaneous detection of organophosphorus pesticides based on an mAb with broad specificity. Li et al. [39] developed a chemiluminescence immunoassay for the detection of sulfonamides based on an mAb that simultaneously recognizes 32 sulfonamides.

#### 3.4.3. Evaluation of Stability

**Figure 11:**
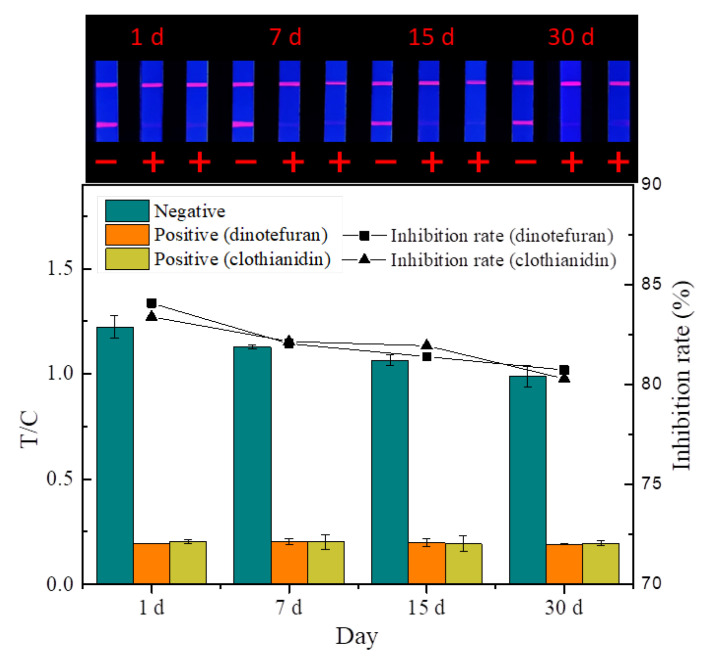
Stability test (*n* = 3).

The quality of the prepared probes and strips is affected by temperature and humidity during storage. The stability experiment (Figure 11) showed that the fluorescence intensity and inhibition of the strip decreased slightly at room temperature under dry conditions after 30 days, indicating that the TRFMs-LFIA established has good stability.

#### 3.4.4. Evaluation of Accuracy

1. Elimination of matrix effect

**Figure 12:**
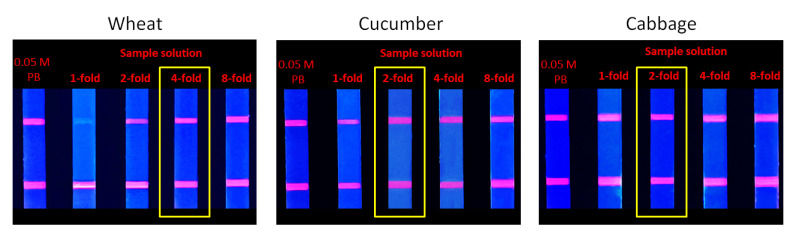
Elimination of matrix effect.

In this study, three samples were selected in the form of wheat, cucumber, and cabbage, which had a relatively complex composition. These diverse components can affect the detection, which is called the matrix effect. The elimination experiment of matrix effect demonstrated that the fluorescence intensities of the 4- and 2-fold dilution of the extract obtained from the wheat, cucumber, and cabbage samples were almost consistent with the control group (Figure 12), which proved that the matrix effect was basically eliminated.

In addition, this study also tested QuEChERS as a pretreatment method. The fluorescence intensity of the 2-fold dilution of the extract obtained from wheat, cucumber, and cabbage samples was almost consistent with the control group. However, QuEChERS needs large instruments and a variety of reagents, making the process complicated, time-consuming, and expensive. In contrast, the pretreatment method described in this study is more convenient, rapid, and more suitable for field detection.

2. Recovery test

As shown in Table 3, the recovery test found that the recovery rates and the corresponding CV ranged from 88.0% to 112.7% and from 1.9% to 12.3%, respectively, in the samples. In addition to the pretreatment method in Table 2, the study also tested QuEChERS, and the recovery rates and the corresponding CV ranged from 89.2% to 114.8% and from 2.9% to 13.5%, respectively, in the samples. Both methods are suitable for sample pretreatment.

### 3.5. Real Sample Detection

The actual wheat, cucumber, and cabbage samples were tested with TRFMs-LFIA, and the results were in agreement with UPLC-MS/MS (Table 4), which shows that the established TRFMs-LFIA can accurately quantify the total content of dinotefuran and clothianidin, and the results agree with the instrumental method. Instrumental methods can achieve accurate qualitative and quantitative analysis but are not applicable for rapid field detection. The TRFMs-LFIA established in this study is simple and quick (lasting about 30 min including pretreatment) and suitable for rapid field detection. However, in actual tests, the complex components contained in the sample, such as lipids, proteins, polysaccharides, and inorganic salts, can affect the test, leading to the generation of false positive or false negative results. To avoid these disturbances, different samples need to be tested separately for the elimination of matrix effects [40].

## 4. Conclusions

In conclusion, a highly sensitive and time-saving TRFMs-LFIA based on mAbs for the simultaneous detection of dinotefuran (IC_50_ = 0.61 ng/mL) and clothianidin (IC_50_ = 0.94 ng/mL) was established in this study. The haptens were synthesized by simpler steps than those reported for similar haptens [17]. With a time-consuming pretreatment method, the detection is more rapid (about 30 min, including pretreatment) than the previously reported immunoassay [15]. The TRFMs-LFIA is stable and accurate, making it suitable for real sample detection.

The TRFMs-LFIA established in this study can be used for the primary screening of dinotefuran or clothianidin in actual detection, making the detection more rapid and simple, and the results can be further accurately verified using instrumental methods. At the same time, the method established in this study still has some limitations that need to be addressed through further research and improvements in the future. For instance, the detection flux and efficiency can be enhanced by integrating sensors [41], microfluidic technology [42,43], or automation [44]. Additionally, similar technical approaches may be explored to develop specific or broad-spectrum antibodies for other nicotinic pesticides, thereby expanding our detection capabilities.

## Figures and Tables

**Table 2:** Cross-reactivity (CR) of dinotefuran with structural analogs.

Analytes	Structure	IC_50_ (ng/mL)	CR (%)
Dinotefuran	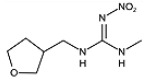	0.61	100.0
Clothianidin	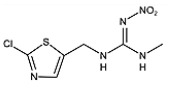	0.94	-
Tetrahydrofuran	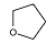	>1000	<0.1
Diuron	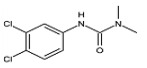	>1000	<0.1
Acetamiprid	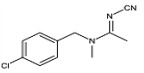	>1000	<0.1
Nitenpyram	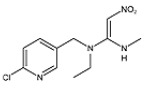	>1000	<0.1
Thiacloprid	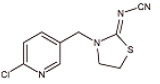	111.70	0.6
Thiamethoxam	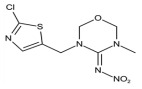	>1000	<0.1
Imidacloprid	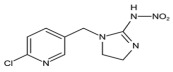	50.82	1.2

**Table 3:** Results of recovery test (*n* = 3).

Samples	Pesticide Standard	Additive Amount (ng/g)	Level in Dilution (ng/mL)	Mean ± SD (ng/mL)	Recovery Rates (%)	CV (%)
Wheat	Dinotefuran	2	0.25	0.259 ± 0.02	103.6	7.7
		8	1	1.058 ± 0.02	105.8	1.9
		24	3	3.051 ± 0.26	101.7	8.5
	Clothianidin	2	0.25	0.274 ± 0.01	109.6	3.6
		8	1	1.062 ± 0.06	106.2	5.6
		24	3	3.380 ± 0.24	112.7	7.1
Cucumber	Dinotefuran	1	0.25	0.266 ± 0.03	106.4	11.3
		4	1	1.078 ± 0.03	107.8	2.8
		12	3	2.679 ± 0.30	89.3	11.2
	Clothianidin	1	0.25	0.220 ± 0.01	88.0	4.5
		4	1	1.009 ± 0.04	100.9	4.0
		12	3	2.688 ± 0.31	89.6	11.5
Cabbage	Dinotefuran	1	0.25	0.233 ± 0.01	93.2	4.3
		4	1	0.980 ± 0.03	98.0	3.1
		12	3	2.772 ± 0.34	92.4	12.3
	Clothianidin	1	0.25	0.249 ± 0.01	99.6	4.0
		4	1	0.982 ± 0.05	98.2	5.1
		12	3	2.691 ± 0.30	89.7	11.1

**Table 4:** Real sample detection results.

Samples	Number	TRFMs-LFIA (ng/g)	UPLC-MS/MS (ng/g)	
		Calculated by Dinotefuran	Dinotefuran	Clothianidin
Wheat	1	ND *	ND	ND
	2	ND	ND	ND
	3	17.87	ND	18.65
	4	14.63	4.55	10.39
Cucumber	1	ND	ND	ND
	2	ND	ND	ND
	3	6.93	ND	7.5
	4	18.35	ND	19.91
Cabbage	1	ND	ND	ND
	2	2.25	ND	2.93
	3	21.29	ND	23.4
	4	8.57	4.12	3.43

* ND, not detectable. Samples numbered 1 were the negative control samples.

## Data Availability

The original contributions presented in the study are included in the article, further inquiries can be directed to the corresponding author.
